# Synthetic and real data sets for benchmarking non-cryptographic hash functions

**DOI:** 10.1016/j.dib.2019.104046

**Published:** 2019-05-22

**Authors:** Yago Saez, Cesar Estebanez, David Quintana, Pedro Isasi

**Affiliations:** a Universidad Carlos III de Madrid, Computer Science Department, Avda. de la Universidad 30, Leganés, Madrid, Spain; b Tuenti, Software Development Department, Madrid, Spain

**Keywords:** Hash, Non-cryptographic hash functions, Benchmark, Hashing datasets

## Abstract

The overall assessment of non-cryptographic functions is very complex and there is not a widely used benchmark. These data have been collected and created as a benchmark for testing non-cryptographic hash functions. It is made up of eight dataset which comes from two different groups, real and synthetic data sources. The objective when selecting and generating the data has been redundancy and structures present in real-world scenarios. These data have been used for benchmarking non-cryptographic hash functions in [1] and [2].

**Table:** Specifications table [please fill in right-hand column of the table below]

Subject area	*Computer Science*
More specific subject area	*Hash functions*
Type of data	*Text files*
How data was acquired	*Manually collected and programmatically generated. Code was developed in C and Java, and executed in a Intel Core 2 Duo (2.*8GHz*) with* 4 GB RAM*, under a OS X Snow Leopard.*
Data format	*Raw*
Experimental factors	*In 2015 the authors searched for publicly available dataset for comparing non-cryptographic hash functions. After having conducted an exhaustive literature research there were not a single unified dataset for comparing this type of functions. Based on this research we started designing a dataset for this purpose.*
Experimental features	*These data sets have been selected to comply with at least with one of the patterns described by Jenkins*[3]. *In addition, for this selection, we have followed the recommendations of Fai*[4]*for desirable features in datasets for testing non-cryptographic hash functions.*
Data source location	
Data accessibility	*Data is available with this article*
Related research article	These data have been used in these 2 research articles: 1 Saez, Y., Estebanez, C., Quintana, D., Isasi, P. (2019), Evolutionary hash functions for specific domains, Applied Soft Computing Journal 78, 58–69. 2 Estebanez, C., Saez, Y., Recio, G., and Isasi, P. (2014), Automated Design of Noncryptographic hash functions using genetic programming, Computational Intelligence, 4, 798–831.

**Table:** 

**Value of the data**. • These data sets are useful for other researchers because they can be used as benchmark when trying to develop and compare the performance of Non-Cryptographic Hash Functions (NCHFs). • All researchers developing new hash functions can benefit from these data, that offers a diverse data collection that can be used as standard benchmark. • The collected data is heterogeneous and have been carefully selected to represent diverse circumstances that can be challenging for NCHFs. • Researchers working with hash functions can focus their effort on designing better functions instead of wasting time with the datasets. • These data sets can be used entirely to test hash functions quality measuring collision resistance, output distributions, avalanche effect or speed [5], [6]. It can be also used for applying machine learning techniques for automated generation by splitting them into training and test sets.

## 1 Data

For creating and selecting these data, we have followed the recommendations of Jenkins and Fai for desirable features in datasets for testing NCHFs [3], [4].

We have put together 8 different data sets coming from two sources [1]:

• **Synthetic data**: data sets that are automatically generated using different distribution functions, see Table 1.

**Table 1:** Synthetic data description.

File name	Description	Size
synth.RANDOM.10000x128.txt	Bits are randomly created following a uniform distribution. This set of data does not contain regularities or any given structure	1000 elements of 128 bits
synth.SPARSE.10000x128.txt	Bits have been sampled from an unbalanced random distribution, in which the probability that a bit is 1 is 10%	1000 elements of 128 bits
synth.REPEAT.10000x512.txt	Natural languages contain redundancies and repeated structures. These words have been drawn from the 16 most frequent English words	1000 vectors of 512 bits
synth.VARIABLE.10000x512.txt	This data set contains only ‘a’ character with some blank spaces (with proportion of 10%), inputs are quite similar	Variable sizes between 80 and 512 bits to force the keys to differ only in length and number of spaces
• **Real data**: data sets extracted from real-world cases, see Table 2.

**Table 2:** Synthetic data description.

File name	Description	Size
real.lcc-keys.txt	Hash functions are an important part of lexical analyzers and compilers. This data set includes all the symbols when compiling the source code of the *lcc* ANSI C compiler [7]	62,901 elements variable length
real.names.noHeader.txt	This is a common real-world data set. It is a collection of all the names allowed by the Argentine government for newborns. In addition to the names, the dataset contains supplementary information such as sex, origin, meaning, etc. This set has the peculiarity of being formed, to a large extent, by repeated or very similar HTML tags, which reduces the amount of information present, increasing its redundancy.	9686 elements with variable length
real.passwords.txt	As a representation of short alphanumeric vectors, very common in hashing applications, we have used a set of very frequent passwords in 13 different languages	3,721,255 elements with variable length
real.megatab.todo.txt	This key set has been extracted from an 18 GB MySQL table with 100,000,000 rows, each of which contains 26 different data fields for a person: complete name, id number, gender, age, and so on. To construct this key set, we randomly extracted 2000 records from the table and only used the following columns: first name, middle name, last name, nationality, gender, and age.	2000 records with variable size

When using these data, NCHFs can be applied with different padding schemes, load factors, and table sizes [2], but we recommend always using them equally for sake of consistency when comparing performances. In the case of using these data sets for generating new NCHFs with machine learning techniques, then they are split into training and test datasets in a random 70%–30% proportion, a minimum 5 k-fold is recommended for cross validation. Only test data set can be used for comparing results.

## 2 Experimental design, materials and methods

All data sets are plain text files with one row per hashed item, Table 1 shows the name and description of each file.
