# Supplementary material for: Synthetic and real data sets for benchmarking non-cryptographic hash functions
Source: Data Brief. 2019 May 22;25:104046. doi: 10.1016/j.dib.2019.104046 (PMC6582058; doi:10.1016/j.dib.2019.104046)
Supplement: Multimedia component 1 [file mmc1.pdf]

I certify that all authors have seen and approved the final version of the manuscript being submitted. They warrant that the article is the authors' original work, hasn't received prior publication and isn't under consideration for publication elsewhere.

I also certify there is no conflict of interest with the generated data, no financial interest or funding, and not potential conflicts with any of the authors.

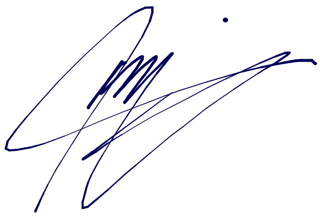

Yago Saez
